# The Southern World

**Published:** 1882-01-20

**Authors:** 


THE SOUTHERN WORLD—a journal for the Farm, Home and Workshop. Published in Atlanta, Georgia, at $1.00 per annum. This is an excellent paper. Its several departments of Agricultural, Stock, Workshop, Home Circle, Young Folks, Sabbath Hour Questions Asked and Answered, are ably conducted by different writers, giving instruction, miscellany, variety, spice and interest adapted to all classes of readers. Every copy beautifully illustrated. It is published twice a month by The Southern World Publishing Company, Atlanta, Ga.

We learn that W. G. Whidby, a good writer and experienced newspaper man of Atlanta, has been recently engaged as business manager of this paper.

The *Southern World* will be sent as a premium to any one who will send us a new subscriber enclosing our regular subscription price, $2.00.

